# Global Prevention of Environmental and Occupational Cancer

**DOI:** 10.1289/ehp.1103871

**Published:** 2011-07-01

**Authors:** Philip J. Landrigan, Carolina Espina, Maria Neira

**Affiliations:** Mount Sinai School of Medicine,, New York, New York, E-mail: phil.landrigan@mssm.edu; World Health Organization, Geneva, Switzerland

Cancer has become the second leading cause of death worldwide (Ferlay et al. 2008). Almost 13 million persons are diagnosed each year with cancer, and 7.6 million die (Ferlay et al. 2010). Today more than half of all cancers and 63% of cancer deaths occur in low- and middle-income countries (LMICs), a burden that is expected to grow in future years as the “Western lifestyle” spreads and the number of persons in LMICs who live to old age continues to increase (Ferlay et al. 2010).

Toxic exposures in the environment, including workplace exposures, are responsible for a substantial percentage of all cancers (Danaei et al. 2005 Christiani 2011). Precise apportionment is not possible because of gaps in the exposure data, interactions between environmental and lifestyle carcinogens, and differences from country to country in exposure patterns (Prüss-Üstün and Corvalán 2006). However, credible estimates from the World Health Organization (WHO 2009) and the Internation Agency for Research on Cancer (IARC; Straif 2008) suggest that the fraction of global cancer currently attributable to toxic environmental exposures is between 7% and 19%.

Asbestos, silica, arsenic, and radon are among the most common environmental carcinogens. All are considered proven causes of human cancer by IARC (El Ghissassi et al. 2009; Straif et al. 2009). Exposures to all remain widespread and are especially intense and uncontrolled in LMICs. Asbestos, for example, continues to be produced and used in quantities of nearly 2 million tons per year (U.S. Geological Survey 2011). While its use in Western Europe, the United States, and Canada has virtually ceased, export to the developing world is aggressively marketed and steadily increasing (Allen and Kazan-Allen 2008). For example, between 2000 and 2007, India’s consumption of asbestos is reported to have doubled (Burki 2010).

Many cancers caused by environmental and occupational exposures can be prevented (Christiani 2011). Primary prevention—environmental interventions that halt the exposures that cause cancer—is the single most effective strategy. Primary prevention reduces cancer incidence, and it saves lives and billions of dollars. Successful examples include reductions in lung cancer and mesothelioma following bans on asbestos, reductions in bladder cancer after elimination of aniline dyes, reductions in leukemia following imposition of controls on benzene, and termination of hepatic angiosarcoma in chemical workers following introduction of closed-circuit technology for vinyl chloride polymerization (Christiani 2011).

Despite their proven feasibility and cost-effectiveness, efforts to prevent environmental cancers have lagged. In contrast to vigorous and well-coordinated global efforts to prevent cancers caused by tobacco (WHO 2003), much more needs to be done in environmental cancer control and to further develop strategies for prevention of environmental causes of cancer (WHO 2008).

To address these gaps and to develop a new global policy framework for environmental cancer, the WHO convened an international conference on “Environmental and Occupational Determinants of Cancer: Interventions for Primary Prevention” in Asturias, Spain, on 17–18 March 2011. The conference produced the “Asturias Declaration” (WHO International Conference on Environmental and Occupational Determinants of Cancer: Interventions for Primary Prevention 2011), which recommends that primary prevention of environmental and occupational cancers be an integral component of global cancer control. Specific recommendations of the declaration include the following:

- The WHO should develop a global framework for control of environmental and occupational carcinogens that concentrates on the exposures identified by IARC as proven or probable causes of human cancer (IARC 2011).
- The WHO should develop measurable indicators of carcinogen exposure and cancer burden to guide cancer surveillance worldwide.
- All countries need to adopt and enforce legislation and regulations to protect their populations, especially the most vulnerable (pregnant women, fetuses, infants, children, and workers) against environmental and occupational cancers.
- All countries need to develop communication campaigns tailored to local needs to educate their populations about environmental causes of cancer and prevention strategies.
- Corporations should comply with all rules and regulations for prevention of environmental and occupational cancers and adhere to the same standards in all countries—developed and developing—in which they and their subsidiaries operate.

Conference participants agreed that successful prevention of environmental cancer will require partnerships among countries and collaborations of public health authorities with ministries of environment, labor, finance, and trade. In addition, independent, publicly funded research on environmental and occupational causes of cancer is an essential prerequisite to prevention (Tomatis 1995).

The recommendations made by the participants in the “Asturias Declaration” complement and reinforce cancer control strategies focused on individual behaviors and medical practice. They will contribute to prevention of diseases beyond cancer and therefore synergize with the United Nations (UN) global agenda for control of noncommunicable diseases that is to be discussed at the UN General Assembly in September 2011 (UN General Assembly 2010). These recommendations will also prevent recurrence of such tragedies as the global asbestos epidemic, which now claims > 100,000 lives each year.
